# Fructose‐induced salt‐sensitive blood pressure differentially affects sympathetically mediated aortic stiffness in male and female Sprague‐Dawley rats

**DOI:** 10.14814/phy2.15687

**Published:** 2023-05-09

**Authors:** Dragana Komnenov, Noreen F. Rossi

**Affiliations:** ^1^ Department of Physiology Wayne State University Detroit Michigan USA; ^2^ John D. Dingell VA Medical Center Detroit Michigan USA; ^3^ Department of Internal Medicine Wayne State University Detroit Michigan USA

## Abstract

Hypertension is the leading risk factor for major adverse cardiovascular events (MACE). Aortic stiffness and sympathoexcitation are robust predictors of MACE. Combined high fructose and sodium intake increases arterial pressure, aortic stiffness, renin, and sympathetic nerve activity in male rats. We hypothesized that activation of the renin angiotensin system (RAS) and/or the sympathetic system mediates aortic stiffness in rats with fructose‐induced salt‐sensitive blood pressure. Male and female Sprague‐Dawley rats ingested 20% fructose or 20% glucose in drinking water with 0.4% NaCl chow for 1 week. Then, fructose‐fed rats were switched to 4% NaCl chow (Fru + HS); glucose‐fed rats remained on 0.4% NaCl chow (Glu + NS, controls for caloric intake). After 2 weeks, mean arterial pressure (MAP) and aortic pulsed wave velocity (PWV) were evaluated at baseline or after acute intravenous vehicle, clonidine, enalapril, losartan, or hydrochlorothiazide. Baseline global longitudinal strain (GLS) was also assessed. MAP and PWV were greater in male Fru + HS versus Glu + NS male rats (*p* < 0.05 and *p* < 0.001, respectively). PWV was similar between the female groups. Despite similarly reduced MAP after clonidine, PWV decreased in Fru + HS versus Glu + NS male rats (*p* < 0.01). Clonidine induced similar decreases in MAP and PWV in females on either diet. GLS was lower in Fru + HS versus Glu + NS male rats and either of the female groups. Thus, acute sympathoinhibition improved aortic compliance in male rats with fructose salt‐sensitive blood pressure. Female rats retained aortic compliance regardless of diet. Acute RAS inhibition exerted no significant effects. Male rats on fructose high salt diet displayed an early deficit in myocardial function. Taken together, these findings suggest that adult female rats are protected from the impact of fructose and high salt diet on blood pressure, aortic stiffness, and early left ventricular dysfunction compared with male rats.

## Graphical abstract

In male rats, fructose‐high salt diet results in elevated blood pressure and increased aortic stiffness. Despite earlier evidence of preserved left ventricular ejection fraction (LVEF), male rats on the fructose high salt diet display a deficit in myocardial function measured as reduced global longitudinal strain. Acute sympathoinhibition, but not RAS antagonism improves blood pressure and aortic stiffness in males. Female rats fed the same fructose‐high salt diet maintained normal blood pressure, aortic compliance, and global longitudinal strain comparable to those measured in their control glucose‐fed counterparts.

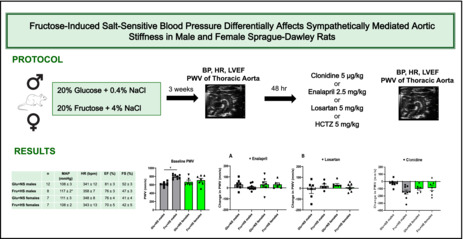

## 1 INTRODUCTION

Arterial stiffness is an independent risk factor for the development and progression of cardiovascular disease (CVD) and development of major adverse cardiovascular events (MACE) (Abboud & Huston, 1961; Laurent et al., 2006; Mitchell, 2008; Mitchell et al., 2010). Although an increase in vascular stiffness with aging is a physiological phenomenon, the presence of hypertension, diabetes, or obesity greatly accelerates the process and decreases the age of onset (Cavalcante et al., 2011). Importantly, these conditions are strongly correlated with consumption of a western diet consisting of high fructose, salt, and fat (Johnson et al., 2007; Khitan & Kim, 2013; Sowers, 2013). Progress in implementing healthy dietary habits has proven too slow and costly with socioeconomic disparities adding to the challenges (Althoff et al., 2022; Jankovic et al., 2021; Thorndike et al., 2022). Such associations highlight the importance of vascular stiffness not only as a diagnostic factor, but also as a therapeutic target in CVD and associated comorbidities.

Aortic pulse wave velocity (PWV), the velocity at which the pressure wave propagates from one point to another within the aorta, is an indirect measure of aortic stiffness. As such, PWV has been used as a marker of aortic stiffness in vivo and can be reassessed over time as well as after therapeutic interventions. Increased arterial stiffness has been observed in hypertensive patients and normotensive subjects predisposed to develop hypertension (Femia et al., 2007). Notably, elevated PWV in normotensive individuals predicts the development of incident elevations in both systolic and diastolic blood pressure 4 years later (Koivistoinen et al., 2018). The implications are at least twofold. First, measuring arterial stiffness may unmask cardiovascular dysfunction well before other parameters, such as blood pressure, become overtly deranged. Second, aiming therapeutic strategies at decreasing arterial stiffness may attenuate downstream perturbations of cardiovascular function. This is especially pertinent to sex‐based differences of CVD incidence. Lean non‐diabetic adult women have lower CVD incidence compared to age‐matched men. However, obese or diabetic females are at greater risk of developing arterial stiffness compared to age‐matched men and experiencing their first MACE episode (Berry et al., 2004; Pal & Radavelli‐Bagatini, 2013).

The factors leading to increased arterial stiffness, with or without overt hypertension, in non‐aged individuals have only recently come to light. In young adult rats, a diet rich in fructose and high in salt increases PWV and decreases aortic distensibility, a direct index of compliance (Komnenov et al., 2020; Levanovich et al., 2021). Similar to humans with prediabetes (Gordish et al., 2017; Sm et al., 2022), rats ingesting a 20% fructose diet exhibit normal serum glucose (Gordish et al., 2017) but clearly demonstrate insulin resistance (Komnenov et al., 2020; Soncrant et al., 2018).

Notably, combined high fructose and high salt intake results in elevated renal sympathetic nerve activity (Gordish et al., 2017; Soncrant et al., 2018). Renal denervation decreases arterial pressure and improves insulin sensitivity, but the impact of sympathoinhibition on vascular stiffness has received less attention. Emerging data show that sympathetic inputs directly impact aortic stiffness. Thus, the sympathetic nervous system (SNS) can be a key regulator of vascular function, and is capable of modulating mechanical properties and function not only of resistance arteries but also of large conduit arteries. This relationship has been confirmed by the studies done in humans showing an independent link between PWV and muscle sympathetic nerve activity in healthy individuals (Swierblewska et al., 2010). Moreover, sympathetic activation results in maladaptive phenomena capable of producing lasting changes via vascular remodeling (Grassi et al., 2009; Holwerda, Luehrs, DuBose, Collins, et al., 2019) independent of arterial pressure (Nardone et al., 2018). As a result, other coefficients of vascular function such as blood pressure become dysregulated. Heightened SNS activity can further induce increases in blood pressure via several mechanisms: inducing peripheral vasoconstriction, potentiating cardiac contraction via cardiac efferent inputs, modulating sodium and water reabsorption by the kidney, and inducing baroreflex dysfunction (Fink & Arthur, 2009). Thus, both acute (Casey et al., 2011) and chronic (Bruijns et al., 1998) sympathetic stimulation can directly or indirectly contribute to increasing vascular stiffness. The latter was demonstrated in acutely sympathectomized rats which show an increase in vascular compliance (Lacolley et al., 1995). Importantly, surgical thoracic sympathectomy causes structural and functional remodeling of the aorta in a porcine model (Angouras et al., 2012). Intriguingly, PWV is predictive of the systolic blood pressure decline in response to renal denervation in hypertensive human subjects (Fengler et al., 2017). Recent data indicate that sex and age influence the impact of sympathoexcitation on arterial elasticity (Holwerda, Luehrs, DuBose, Majee, & Pierce, 2019). Overall, diseases, environmental influences, and dietary habits which heighten sympathetic traffic may increase vascular stiffness. Since male rats fed a fructose and high salt diet have increased PWV (Komnenov et al., 2020; Levanovich et al., 2021), the present study was designed to evaluate whether acute pharmacological sympathoinhibition would improve their aortic stiffness.

Rats ingesting a high fructose and high salt diet also fail to suppress plasma renin activity despite elevated arterial pressure (Gordish et al., 2017; Levanovich et al., 2021). Various studies have shown that arterial stiffness is associated with inappropriate activation of the renin angiotensin system (RAS) (Aroor et al., 2013; Kiuchi et al., 2015; Koumaras et al., 2014; Mahmud & Feely, 2004; Neves et al., 2018; Shapiro et al., 2008). In addition to classically activated RAS and its circulating components, tissue components of RAS may also become recruited within the vasculature during various pathophysiologic states (Dzau & Re, 1994; Hanna et al., 2002; Hilgers et al., 2001; Schulman et al., 2005; Zhuo et al., 1998). Within vascular tissue, angiotensin II (Ang II) can be formed not only by angiotensin converting enzyme (ACE) but also via chymases and cathepsins (Bader, 2010; Kumar et al., 2012; Lavrentyev et al., 2007; Richard et al., 2001). Vascular Ang II, in turn, can induce formation of reactive oxygen species which are increased in male fructose‐fed rats (Komnenov et al., 2020; Zenner et al., 2018). In addition, sex differences have been observed in the cardiovascular responses to both classic and tissue RAS (Fischer et al., 2002). Females exhibit lower expression of AT1 receptor (AT1R) thus dampening the action of Ang II (Maric‐Bilkan & Manigrasso, 2012; Yung et al., 2011) and decreasing inflammatory responses (Meyer et al., 2011; Ribas et al., 2011). As an initial screen, we deemed it reasonable also to assess the impact of acute pharmacological inhibition of RAS on aortic stiffness in the rat model of fructose high salt diet.

The impact of a diet consisting of high fructose plus high fat, but not high salt, for extended periods of time (i.e., 16 weeks) on aortic stiffness and vascular dysfunction has been studied in female mice and demonstrated increases in vascular stiffness (Jia et al., 2016; Lastra et al., 2017). Paradoxically, pharmacological interventions inhibiting oxidative stress in female mice improved vascular compliance measured in vitro, yet PWV measur*ed* in vivo *remained* elevated and comparable to that in untreated female mice (Lastra et al., 2017). An identical pattern of response occurred in female mice fed high fructose plus high fat diet for 16 weeks when exercise was used as a therapeutic modality (Padilla et al., 2016). The impact of the fructose‐high salt diet on blood pressure and vascular stiffness and pharmacologic intervention in female rats has not been assessed. (Soncrant et al., 2018).

A diet with 20% fructose and high salt for 3 weeks in male rats leads to elevated blood pressure, increased aortic stiffness and diastolic left ventricular (LV) dysfunction while causing no change in LV systolic function measured as ejection fraction (Komnenov et al., 2020). Among other limitations, LV ejection fraction is dependent on loading conditions whereas global longitudinal strain (GLS) is a more sensitive marker of systolic LV function (Klaeboe & Edvardsen, 2019; Stanton et al., 2009). Thus, GLS may unmask more subtle differences in LV systolic function in the fructose‐high salt diet rodent model.

In the present investigation, we hypothesized that acute pharmacological inhibition of sympathetic output but not RAS will improve aortic stiffness caused by a diet with 20% fructose and 4% sodium chloride in Sprague‐Dawley rats and that GLS will reveal abnormal systolic LV function not apparent with LV ejection fraction. Additionally, we hypothesized that a sex‐based dichotomy will be evident in both the arterial pressure and vascular properties assessed by ultrasound on these outcomes.

## 2 METHODS

### 2.1 Animals

Male and female Sprague‐Dawley rats (Envigo, Indianapolis, IN) at 11–12 weeks of age were housed under controlled conditions (21–23°C; 12 h light and 12 h dark cycles, light on at 6 am) and were permitted ad libitum access to water and standard rat chow containing 0.4% NaCl until they were enrolled into experimental protocols described below. Complete care provided to rats was in accordance with the principles of the National Research Council Committee *Guide for the Care and Use of Laboratory Animals*. All procedures and protocols were approved by the Wayne State University Institutional Animal Care and Use Committee. Since our previous experiments demonstrated no difference in PWV or left ventricular diastolic dysfunction in glucose plus high salt or fructose plus normal salt diet compared with glucose plus normal salt control rats (Komnenov et al., 2020), we adhered to the principle of reducing the number of rats by testing only fructose plus high salt rats compared with glucose normal salt controls.

### 2.2 Dietary regimen and surgical procedures

Upon arrival, all rats were provided ad libitum access to 0.4% NaCl standard chow (Envigo Teklad) and normal water and were permitted to acclimate to the vivarium for 5 days. Then, the rats were randomized to either 20% (w/v) glucose (Sigma‐Aldrich) or 20% (w/v) fructose (Sigma‐Aldrich) in their drinking water and maintained at 0.4% NaCl chow (normal salt) for 1 week. After 1 week, the rats were maintained on glucose and 0.4% salt (Glu + NS) and the fructose‐fed rats were placed on 4% NaCl chow (high salt; Fru + HS). This dietary paradigm was continued for two additional weeks. Glucose was provided in the control group to match both caloric and fluid intake as previously done in male rats (Komnenov et al., 2020).

The rats were instrumented with carotid and jugular catheters at the beginning of week four. Briefly, rats were anesthetized with a ketamine/xylazine cocktail (80/10 mg/kg, respectively), and analgesia was provided with sustained release buprenorphine (0.05 mg/kg) given subcutaneously before the start of the procedures. A midline incision was made in the ventral neck and the left carotid artery exposed using blunt dissection. Blood flow was attenuated with a vessel clip and arterial catheter inserted through a small incision made with a pair of micro‐scissors. The catheter was secured in place with three sutures. The internal jugular vessel was catheterized in a similar manner. The catheters were tunneled and exteriorized at the base of the neck, and the incision was sutured. The rats were allowed to recover for 1 day after which the experimental procedures were initiated as we described before (Komnenov et al., 2020; Levanovich et al., 2021).

### 2.3 2‐D Echocardiography and speckle tracking echocardiography

Hemodynamic measurements, echocardiography, and speckle tracking echocardiography were completed by a the same investigator (DK) blinded to the group assignments.

Rats were anesthetized with 3% isoflurane in an induction chamber, and maintenance of anesthesia was achieved with 1.5% isoflurane delivered via a nose cone. Scans were performed on the heated platform (Fujifilm Visualsonics; Inc.) in a supine position with all four limbs secured to ECG electrodes with tape. Fur was removed from the chest and abdominal areas first by shaving the area followed by a cream depilatory (Pharmaceutical Innovations), before applying contact gel preheated to 37°C. Body temperature was monitored using a rectal probe. Blood pressure and heart rate were obtained via arterial catheter and monitored continuously with PowerLab connected to the LabChart 8.0 software (ADInstruments). Echocardiographic recordings were made according to standard methods at the conclusion of the dietary intervention. Images were acquired using a Vevo 3100 ultrasound instrument with an MX‐250S transducer (Fujifilm Visualsonics Inc.). A parasternal short axis view was used to obtain images in M‐mode at the level of papillary muscles to assess systolic function and left ventricular dimensions. Left ventricular diastolic function was ascertained using pulse wave Doppler recordings of transmitral flow velocities aligned in the apical four chamber view. Aortic PWV was determined using B‐mode and pulse wave Doppler of aortic flow velocities at two positions along the aortic arch. Briefly, the time to initiation of flow was determined relative to the ECG and the difference calculated. The distance between the two flow positions, determined along the B‐mode image, was then divided by the time to calculate the PWV. Speckle tracking was completed on images obtained in B‐mode in the parasternal view. Strain measurements were obtained by off‐line analysis using VevoLab software with the Vevo Strain module.

### 2.4 Acute inhibition of RAS, SNS, or renal sodium excretion

The choice of agent tested first was determined randomly. Some rats were able to be tested with more than one intervention. At least 48 h (>5 half‐life of the drugs) elapsed between test periods to reduce any residual effects of the previous agent. Dosages were chosen based on the literature to try to achieve comparable changes in MAP among the agents. All testing was done between 10 am and 12:00 pm.

Once the rat was anesthetized as above, it was permitted to equilibrate for 10 ± 2 min and baseline measurements for arterial pressure, heart rate, PWV, and GLS were obtained. To assess the impact on aortic PWV a bolus injection of one of the following agents was then given intravenously and PWV reassessed after the MAP stabilized (5 ± 1 min): angiotensin converting enzyme (ACE)‐inhibitor enalapril (2.5 mg/kg), an angiotensin receptor blocker (ARB) losartan (5 mg/kg) (Maliszewska‐Scislo et al., 2003), and the sympathoinhibitor clonidine (5 μg/kg) (Takishita et al., 1994). Since thiazide diuretics are recommended as a first line antihypertensive medication (Whelton et al., 2018), we also performed these measurements 5 min after a single dose of the diuretic hydrochlorothiazide (5 mg/kg i.v.) (Tanoue et al., 1982).

### 2.5 Analyses and statistics

Analysis of hemodynamic parameters and echocardiographic data were performed by a researcher blinded to the group assignments. The study was powered for PWV based on previously published values in this model (Komnenov et al., 2020; Levanovich et al., 2021) such that with a standard deviation of 40, five observations per group would permit finding a difference of 100 mm/s with a power of 90% at an α level of 0.05. Data are presented as the mean ± SE. Comparisons in the same animal in a given group between baseline and post‐drug were accomplished by paired *t*‐test. Comparisons among groups were completed using two‐way ANOVA followed by Holm–Ṡidak post hoc test when appropriate. A *p‐*value <0.05 was taken as significant.

## 3 RESULTS

### 3.1 Baseline hemodynamics, PWV, and LV function

Rats were 15 ± 1 weeks of age at the end of the experiment. Sex (*F* = 24.47; *p* < 0.0001) but not diet (*F* = 1.20; *p* = 0.35) had an impact on body weight. Male rats had similar body weight regardless of diet: 327 ± 11 g (*n* = 12, Glu + NS) and 327 ± 8 g (*n* = 8, Fru + HS). Both groups of female rats had significantly lower body weights than the male rats in either group (*p* < 0.0001) but did not differ from each other with respect to diet: 258 ± 8 g (*n* = 7, Glu + NS) and 239 ± 3 g (*n* = 7, Fru + HS). Baseline mean arterial pressure (MAP) and heart rate (HR) for the male and female rats on either Glu + NS or Fru + HS diet are shown in Table 1. Sex accounted for the difference in MAP (*F* (1.36) = 8.12; *p* = 0.0072). Furthermore, Fru + HS feeding in males led to an increase in MAP compared to Glu + NS fed rats (*p* < 0.001) as previously observed (Gordish et al., 2017; Komnenov et al., 2020; Soncrant et al., 2018; Zenner et al., 2018) as well as Fru + HS females (*p* < 0.05). The two female groups, however, displayed similar MAPs and HRs irrespective of the diet they were on. The variability of MAP across experiments (calculated as the variability of day‐to‐day baseline values prior to treatment) was 2.9% ± 0.6% (*n* = 34).

**TABLE 1:** Baseline hemodynamic and systolic left ventricular parameters in male and female rats.

	*n*	MAP (mmHg)	HR (bpm)	EF (%)	FS (%)
Glu + NS males	12	108 ± 3	341 ± 12	81 ± 3	52 ± 3
Fru + HS males	8	117 ± 2*	358 ± 7	76 ± 3	47 ± 3
Glu + NS females	7	111 ± 3	348 ± 8	76 ± 4	41 ± 4
Fru + HS females	7	108 ± 2**	343 ± 13	70 ± 5	42 ± 5

*Note*: Values are mean ± SE.

Abbreviations: EF, left ventricular ejection fraction; FS, fractional shortening; HR, heart rate; MAP, mean arterial pressure.

* *p* < 0.001 versus Glu + NS males

** *p* < 0.05 versus Fru + HS males by two‐way ANOVA with Holm–Ṡisak post hoc testing.

**FIGURE 1:**
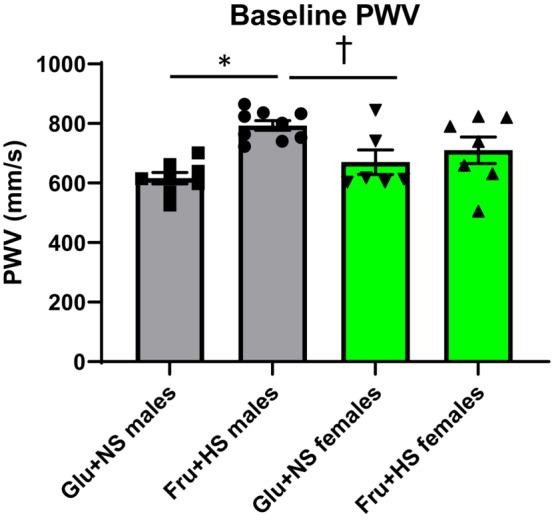
Aortic pulse wave velocity (PWV) in male and female rats fed 20% glucose and 0.4% NaCl chow (Glu + NS) or 20% fructose and 4% NaCl chow (Fru + HS). Values are mean ± SE. *n* = 8, 9, 6 and 7. *p < 0.001 Glu + NS versus Fru + HS males; ^†^ *p* < 0.05 Fru + HS males versus Glu + NS females. Comparisons by two‐way ANOVA with post hoc Holm–Ṡidak comparisons.

Two‐way ANOVA (sex x diet) revealed that there was a statistically significant interaction between sex and diet on PWV (F (1, 26) = 5.159, *p* = 0.032). Simple main effect analysis showed that aortic PWV was higher in male Fru + HS fed rats (793 ± 17 mm/s) compared to the rats on Glu + NS diet (616 ± 19 mm/s, *p* < 0.001; power = 93.3%). No difference in PWV was observed between the two female groups: 710 ± 44 mm/s (Fru + HS) versus 670 ± 41 mm/s (Glu + NS) (Figure 1). No significant difference in aortic PWV was observed between females and males that were on the same diet, but male Fru + HS rats displayed higher PWV than female Glu + NS rats (*p* < 0.05). The day‐to‐day variability of baseline PWV over the 3 days of testing was 2.2% ± 0.4% (*n* = 29).

**FIGURE 2:**
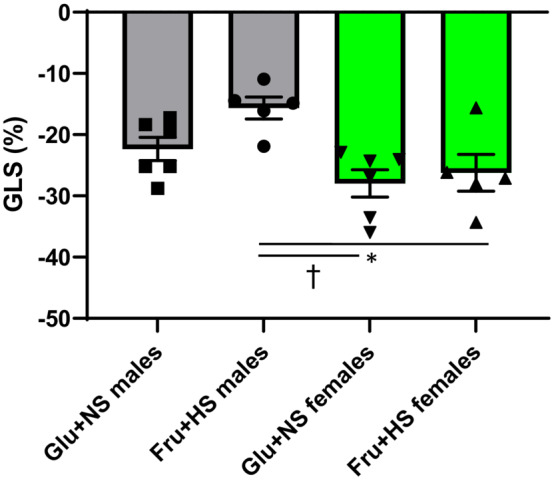
Global longitudinal strain (GLS) in male and female rats fed either fed 20% glucose and 0.4% NaCl chow (Glu + NS) or 20% fructose and 4% NaCl chow (Fru + HS). Values are mean ± SE; *n* is as shown for each observation. *p < 0.01 versus Fru + HS female; ^†^ *p* < 0.01 versus Glu + NS females by two‐way ANOVA with post hoc Holm–Ṡidak comparisons.

Left ventricular ejection fraction was similar between males (76% ± 3% in Fru + HS vs. 81% ± 3% in Glu + NS) and females (70% ± 5% in Fru + HS vs. 76% ± 4% in Glu + NS), irrespective of the diet (Table 1). Similarly, fractional shortening was not altered by diet in either males (47% ± 3% in Fru + HS vs. 52% ± 3% in Glu + NS) or females (42% ± 5% in Fru + HS vs. 41% ± 4% in Glu + NS) (Table 1). GLS measured via speckle tracking echocardiography revealed no significant interaction between sex and diet (*F* (1, 18) = 1.20; *p* = 0.288). Main effects testing showed that sex had a significant effect (*F* (1, 18) = 12.74; *p* = 0.0022): Fru + HS male rats displayed impaired GLS (−15.6% ± 1.8%) compared with female Glu + NS rats (−28.0% ± 2.2%; *p* < 0.03) or female Fru + HS rats (−26.2% ± 3.0%; *p* < 0.01). There was no significant difference in GLS between the female groups. Diet did not account for the variance (*F* (1, 18) = 3.46; *p* = 0.0794) (Figure 2).

### 3.2 Effects of acute blockade of RAS on hemodynamics and aortic PWV

**FIGURE 3:**
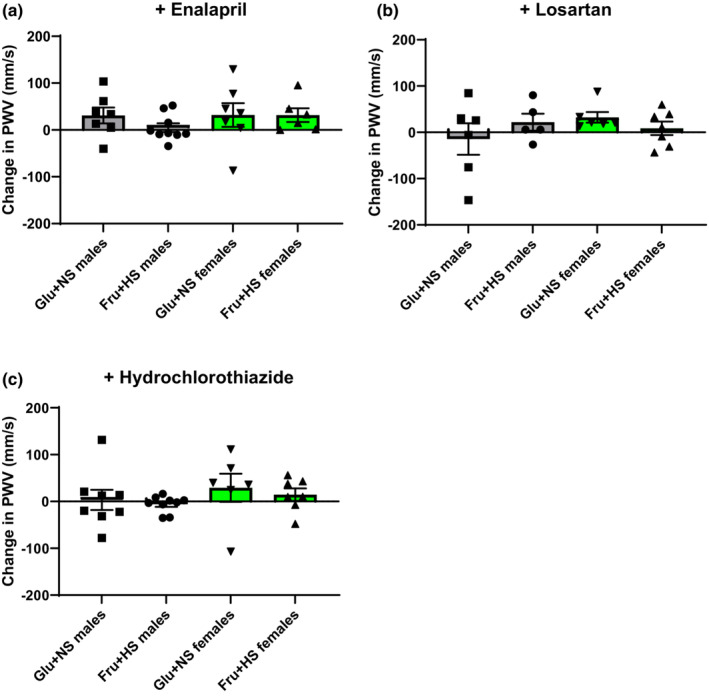
Change in aortic pulse wave velocity (PWV) in male and female rats fed 20% glucose and 0.4% NaCl chow (Glu + NS) or 20% fructose and 4% NaCl chow (Fru + HS) after acute intravenous injection with (a) enalapril, (b) losartan, or (c) hydrochlorothiazide. Values are mean ± SE; *n* is as shown for each observation. No significant changes were observed among the groups with any of the treatments by two‐way ANOVA with post hoc Holm–Ṡidak comparisons.

There was no interaction regarding the decreases in MAP after enalapril administration as shown in Table 2. Main effects analysis revealed a significant impact of diet (*F* (1, 25) = 12.30; *p* = 0.0017) but not sex (*F* (1, 25) = 2.76; *p* = 0.1091). Glu + NS female rats displayed the greatest reduction in MAP which was significantly different from either Fru + HS males or Fru + HS females. There were no differences in the change in HR among the groups. The change in aortic PWV did not display any interaction (*F* (1, 26) = 1.40; *p* = 0.248) from baseline after acute treatment with enalapril in any of the groups, nor any main effects (Figure 3a).

**TABLE 2:** Changes in mean arterial pressure and heart rate after a single i.v. bolus injection of enalapril, losartan or hydrochlorothiazide.

	*n*	Δ MAP + enalapril (mmHg)	ΔHR + enalapril (bpm)	*n*	Δ MAP + losartan (mmHg)	ΔHR + losartan (bpm)	*n*	Δ MAP + HCTZ (mmHg)	ΔHR + HCTZ (bpm)
Glu + NS males	10	−13 ± 1	−2 ± 4	5	−11 ± 3	+19 ± 17	10	+3 ± 1	+5 ± 4
Fru + HS males	8	−8 ± 2*	−5 ± 3	6	−5 ± 2	−4 ± 4	8	0 ± 0	+8 ± 6
Glu + NS females	5	−22 ± 5	−3 ± 11	7	−7 ± 3	−4 ± 4	7	+1 ± 1	+8 ± 6
Fru + HS females	6	−8 ± 4*	−3 ± 7	6	−8 ± 2	−4 ± 4	7	+2 ± 1	−3 ± 3

*Note*: Enalapril, 2.5 mg/kg i.v; losartan, 5 mg/kg i.v; HCTZ 5 mg/kg i.v. Values are mean ± SE.

Abbreviations: HCTZ, hydrochlorothiazide; HR, heart rate; MAP, mean arterial pressure.

* *p* < 0.05 versus Glu + NS females by two‐way ANOVA with Holm–Ṡidak post hoc analysis.

A single bolus administration of losartan exhibited no interaction in the change in MAP (*F* (1, 25) = 1.59; *p* = 0.219) nor HR (*F* (1, 25) = 0.83; *p* = 0.370) and no main effects (Table 2). Similar to enalapril, the change in aortic PWV did not exhibit an interaction (*F* (1, 20) = 1.97; *p* = 0.176) and was not altered from baseline in any of the groups (Figure 3b).

### 3.3 Effects of acute diuretic administration on hemodynamics and aortic PWV

A significant interaction was observed for the change in MAP between diet and sex after HCTZ infusion (*F* (1, 27) = 4.76; *p* = 0.038), but not for HR (*F* (1, 27) = 1.69; *p* = 0.204). There were no significant changes from baseline or among the groups (Table 2). No interaction was observed in the change in aortic PWV after administration of hydrochlorothiazide (*F* (1, 26) = 0.020; *p* = 0.882) (Figure 3c).

### 3.4 Effect of acute blockade of SNS on hemodynamics and aortic PWV

**FIGURE 4:**
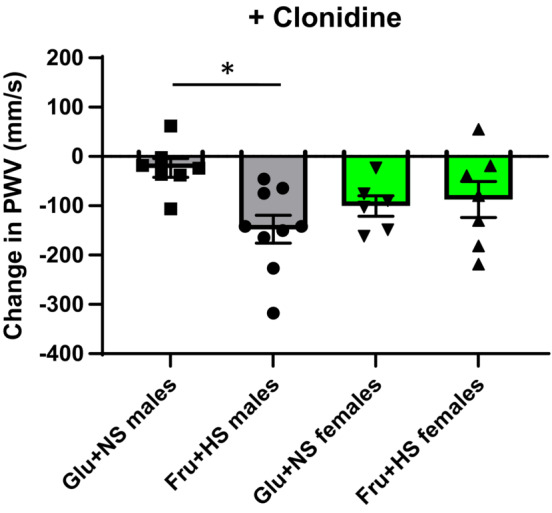
Change in aortic pulse wave velocity (PWV) in male and female rats fed 20% glucose and 0.4% NaCl chow (Glu + NS) or 20% fructose and 4% NaCl chow (Fru + HS) after acute intravenous injection with clonidine. Values are mean ± SE; *n* is as shown for each observation. *p < 0.02 versus Glu + NS males by two‐way ANOVA with post hoc Holm–Ṡidak comparisons.

Table 3 shows the data before and after clonidine injection. Although the MAP itself showed no interaction after clonidine (*F* (1, 25) = 0.80; *p* = 0.3803), post hoc analysis revealed that sex did impact the variance (*F* (1, 25) = 9.77; *p* = 0.0045) with female Glu + NS rats having significantly lower MAP that male Glu + NS rats (*p* < 0.05). The decrease in MAP after clonidine, however, showed no interaction (*F* (1, 25) = 0.55; *p* = 0.467). Neither diet nor sex had an impact on the change in MAP. The decreases in MAP were equivalent in all four groups confirming the design to have equivalent decreases in MAP post treatment. Likewise, HR decreased similarly in all groups after clonidine administration. Baseline aortic PWV immediately prior to the clonidine injection displayed a significant interaction between sex and diet (*F* (1, 25) = 4.66; *p* = 0.041). Diet accounted for a substantial part of the variance (*F* (1, 25) = 13.97; *p =* 0.001*)* but sex did not (*F* (1, 25) = 0.80; *p* = 0.379). Both male Fru + HS and female Fru + HS groups had higher PWV compared with male Glu + NS groups (Table 3, Figure 4). No interaction was identified after clonidine administration (*F* (1, 27) = 0.01; *p* 0.916). There was a main effect of diet (*F* (1, 27) = 5.01; *p* = 0.034) but not sex (*F* (1, 27) = 1.82; *p* = 0.188). A significant interaction between sex and diet was observed in the change in PWV after clonidine (*F* (1, 25) = 5.72; *p* = 0.025). Diet tended to account for the variance (*F* (1, 25) = 3.76; *p =* 0.064) rather than sex (*F* (1, 25) = 0.08; *p =* 0.784). Post hoc analysis showed that the decrease in PWV in male rats fed Fru + HS was greater than in males ingesting Glu + NS (*p* < 0.02).

**TABLE 3:** Mean arterial pressure, heart rate, and aortic pulse wave velocity before and after i.v. bolus injection of clonidine.

	*n*	Before clonidine (mmHg)	After clonidine (mmHg)	Δ MAP + clonidine (mmHg)	HR before clonidine (bpm)	HR after clonidine bmp)	ΔHR + clonidine (bpm)	PWV before clonidine (mm/s)	PWV after clonidine (mm/s)	ΔPWV + clonidine (mm/s)
Glu + NS males	8	108 ± 3	85 ± 2^e^	−23 ± 3	341 ± 12	280 ± 14^Δ^	−61 ± 5	605.3 ± 14.5	582.1 ± 14.9	−23.2 ± 26.9
Fru + HS males	8	112 ± 3^b^ ^,^ ^d^	90 ± 6^e^	−22 ± 3	353 ± 11	293 ± 11^Δ^	−60 ± 6	786.4 ± 19.8^a^	638.2 ± 18.9^a^	−148.2 ± 28.4^a^
Glu + NS females	6	97 ± 3	75 ± 4^a^ ^,^ ^e^	−22 ± 3	332 ± 12	272 ± 2^Δ^	−60 ± 13	644.1 ± 37.1	543.6 ± 34.3^a^ ^,^ ^c^	−100.5 ± 20.6
Fru + HS females	7	99 ± 2	72 ± 5^e^	−27 ± 6	347 ± 17	276 ± 11^Δ^	−71 ± 24	692.6 ± 44.8^a^	605.4 ± 34.7^a^ ^,^ ^d^	−87.2 ± 36.3

*Note*: Values are mean ± SE.

Abbreviations: HR, heart rate; MAP, mean arterial pressure; PWV, aortic pulse wave velocity.

^a^ *p* < 0.05 versus Glu + NS male.

^b^ *p* < 0.05 Fru + HS female.

^c^ *p* < 0.01 versus Fru + HS male.

^d^ *p* < 0.05 versus Glu + NS female by two‐way ANOVA and Holm–Ṡidak post hoc analysis.

^e^ *p* < 0.01 versus MAP before clonidine; Δ *p* < 0.001 versus HR before clonidine by paired *t*‐test.

## 4 DISCUSSION

In our investigation of the mechanisms involved in fructose salt sensitive hypertension in rats, the sympathetic activation rather than RAS is the major driver of the derangements of the cardiovascular function, at least in the acute setting. This is only true for male rats, since adult female rats are afforded cardiovascular protection from dietary fructose, at least in the short‐term (3 weeks) with a moderate amount of dietary fructose. This sexual dichotomy was observed in indices of cardiovascular function such as blood pressure, aortic PWV, and myocardial strain. Male rats on Fru + HS diet had higher MAP and aortic PWV compared to the males on the control diet (Glu + NS) and impaired GLS compared with females on either diet. In fact, the baseline indices were not different between the two female dietary groups. Acute RAS inhibition exerted no impact on PWV despite a greater decrease in MAP in female Glu + NS rats. On the contrary, acute sympathoinhibition with clonidine resulted in equivalent decreases in MAP among all the groups but improved aortic PWV, an index of vascular stiffness, to the greatest extent in male Fru + HS fed rats. The molecular mechanisms behind the protection in female rats are, as of yet, unknown, were not a topic of investigation by the current studies, but warrant further investigation.

### 4.1 Hemodynamic responses to fructose and high salt diet in male versus female rats

Data from our laboratory and those of others have shown that feeding of moderately increased fructose in male Sprague‐Dawley rats causes salt sensitive hypertension within as little as one to 3 weeks (Komnenov et al., 2020; Soncrant et al., 2018). This dietary regimen causes an increase in aortic PWV accompanied by diastolic dysfunction in male rats (Komnenov et al., 2020). The present findings show that the same diet did not induce an increase in either MAP or aortic PWV and did not impair GLS in female rats. It has been demonstrated in both animals and humans that cardiovascular protection against a range of insults is afforded in females. The present study is consistent with female rats being protected from the hemodynamic impact of combined fructose and high salt. This contrasts with recent findings showing female Sprague‐Dawley rats from Charles River display increased systolic blood pressure by tail cuff plethysmography after consuming a diet with similar proportions of fructose and high salt for 1 week (Brostek et al., 2022). It has been shown that Sprague‐Dawley rats from Harlan (incorporated as Envigo in 2015) have a higher basal blood pressure profile than those from Charles River (Pollock & Rekito, 1998) and can display differing results to experimental stimuli (Heimlich & Pollock, 2014). In the present studies, blood pressure was assessed under mild isoflurane anesthesia which may have impacted blood pressure (DeLalio & Stocker, 2021), in contrast to plethysmography which was performed in awake animals (Brostek et al., 2022). Notably, plethysmography assesses only systolic blood pressure. Moreover, habituation to restraint stress required by tail cuff measurements is limited and may disproportionately affect female rats (Mitsushima et al., 2003; Sikora et al., 2016). Furthermore, the blood pressure changes in our male rats are similar to those in earlier reports (Gordish et al., 2017; Komnenov et al., 2020; Soncrant et al., 2018; Zenner et al., 2018). Importantly, the increase in MAP observed in our isoflurane‐anesthetized male Fru + HS rats (~9 mmHg) was similar to that which we observed in a previous study in conscious rats by telemetry (~6 mmHg), which also demonstrated that the difference in MAP was due solely to differences in systolic blood pressure (Komnenov et al., 2020). The impact of timing cannot be discounted as current studies were done at 3 weeks versus 1 week of dietary intake. Thus, the present studies cannot distinguish whether the commercial source of the animals, anesthesia versus tail cuff plethysmography, or duration of dietary manipulation played a factor in the different findings in the female rats, but are consistent with telemetry data in conscious male rats.

### 4.2 Sympathetic mechanisms of aortic stiffness in fructose salt sensitive blood pressure

Present data are in agreement with the hypothesis that fructose plus high salt diet in male rats contributes to heightened sympathetic tone and is at least one mechanism governing impaired systemic hemodynamics and aortic stiffness. Unlike male rats, female rats are protected from increased aortic stiffness and impaired myocardial shortening when fed moderately increased fructose and salt diet. These findings are consistent with studies in humans showing an association of heightened muscle sympathetic tone and aortic stiffness in men but not in women (Casey et al., 2011). Potential mechanisms of this protection include increased sensitivity or upregulation of beta adrenergic signaling (Kneale et al., 2000), altered metabolism of norepinephrine by vascular smooth muscle (Masi et al., 2009), increased endothelial nitric oxide generation (Miller & Duckles, 2008), or a direct effect of estrogen on vasculature in females. This apparent protection from elevated PWV and sympathetic tone has been observed in women and appears to be lost after menopause as evidenced by a greater response to the ganglionic blocker trimethaphan camsylate in older women (Harvey et al., 2017). Investigation of a possible difference in young versus ovariectomized female rats was beyond the scope of the present studies; however, taken together, the present studies and earlier findings support a substantial role for sympathetic nervous system in the augmented aortic stiffness with moderately high fructose and salt feeding as well as a clear sexual dimorphism.

### 4.3 RAS inhibition and diuretic impact on aortic stiffness in fructose salt sensitive blood pressure

The lack of impact on PWV by either angiotensin converting enzyme inhibition or blockade of the AT_1_ receptor may be due to the acute nature of the experimental design. Both types of inhibitors were used at doses designed to obtain a similar drop in MAP as with clonidine and to control for other enzymatic pathways or formation of vasoactive substances such as bradykinin (Bader, 2010; Kumar et al., 2012; Lavrentyev et al., 2007; Richard et al., 2001). However, this was achieved only in the Glu + NS groups. Although not statistically different from the Glu + NS groups except for enalapril, the decrease in MAP was uniformly attenuated in the Fru + HS rats of both sexes. Thus, improvement in PWV may well have been constrained by the more limited reduction in arterial pressure in response to RAS inhibition. Another possible explanation is that the response to acute sympathetic inhibition can be rapidly transduced to the vasculature whereas the mechanisms involving angiotensins, such as vascular remodeling and gene expression, may require more prolonged inhibition to yield a detectable impact on aortic compliance. Likewise, the effect of diuretic clearly was limited in both its effect on arterial pressure and certainly on any anticipated change in extracellular volume during the timeframe of the study.

### 4.4 Global longitudinal strain in fructose salt‐sensitive blood pressure

Identification of left ventricular dysfunction is critical for timely clinical management of cardiovascular disease (Luis et al., 2019). Left ventricular ejection fraction (LVEF) is routinely used to assess myocardial function. Impaired cardiac function with preserved LVEF is a common clinical finding. Subtle changes in left ventricular myocardial function have been reported to occur in increased arterial stiffness (Mottram et al., 2005), affecting myocardial relaxation. Notably, fructose and high salt diet has been shown to decrease left ventricular diastolic dysfunction manifested as a decrease in early to late transmitral filling velocities in male rats (Komnenov et al., 2020). Recently, GLS measured by speckle tracking echocardiography has been used as a more sensitive and reproducible marker of early left ventricular dysfunction (Galderisi et al., 2017; Karlsen et al., 2019; Mor‐Avi et al., 2011) before frankly abnormal of LVEF. Although male rats on the same dietary regimen did not demonstrate a change in LVEF in the current or in previous studies (Komnenov et al., 2020), GLS was decreased in male but not female rats fed fructose and high salt diet. (Karlsen et al., 2019; Komnenov et al., 2020). Moreover, fructose catabolism in cardiac tissue has been identified as a critical pathobiochemical pathway associated with abnormal GLS (Farha et al., 2021). These results highlight the diagnostic and potential prognostic value of PWV and GLS as coefficients of early cardiovascular derangement, before other indices of hemodynamic and cardiovascular function become perturbed in individuals ingesting a fructose‐rich diet and salt sensitive blood pressure.

Indeed, two major meta‐analyses demonstrated that aortic PWV is the most reliable predictor of cardiovascular mortality (Ben‐Shlomo et al., 2014; Vlachopoulos et al., 2010). A recent study in a mixed population of hypertensive and normotensive individuals showed that those in the highest tertile of brachial PWV had the lowest GLS and the highest proportion of hypertensives (54% vs. 12% and 38% in tertiles 1 and 2, respectively) (Duan et al., 2022). Additionally, hypertensive individuals had significantly higher brachial PWV and significantly lower GLS compared to the normotensive participants but this study did not assess any sex‐based dichotomy (Duan et al., 2022). These data agree with studies in male rats with fructose salt sensitive hypertension showing that aortic stiffness is accompanied by a deficit in myocardial shortening as measured by GLS (Duan et al., 2022). The present data indicate that these indices are not deranged in females on fructose and high salt diet, coincides with their failure to increase blood pressure on the fructose high salt diet.

## 5 LIMITATIONS AND FUTURE DIRECTIONS

Although the hemodynamic parameters were obtained via direct recordings from a chronically implanted arterial catheter, the studies were perforce performed under isoflurane anesthesia in order to obtain ultrasound images that rendered themselves to rigorous evaluation of PWV and GLS. The duration and amount of fructose in the diet have varied considerably among earlier studies from 1 week (Brostek et al., 2022; Gordish et al., 2017; Zenner et al., 2018) to several months (Dhar et al., 2013; Katakam et al., 1998). We chose the 20% fructose to approach the amount ingested by the upper quintile of western populations (Bray et al., 2004) and an intermediate time period of 3 weeks when previous studies in males showed elevated blood pressure, PWV, and left ventricular diastolic but not systolic dysfunction as well as normoglycemia but insulin resistance (Komnenov et al., 2020). RAS involvement may require higher dietary fructose or longer exposure to the diet (Dhar et al., 2013; Katakam et al., 1998). Finally, we studied young adult intact male and female rats. Whether gonadal hormones contribute to protection observed in adult female rats (Harvey et al., 2017) or to the derangements in males will required further studies. Regardless, the data suggest that one of the earliest mechanisms reducing aortic compliance and myocardial contractility and relaxation is via the sympathetic nervous system.

## 6 CONCLUSION

In summary, our data indicate that aortic stiffness is sympathetically mediated in male rats with fructose salt‐sensitive blood pressure rather than being under the control of RAS. Furthermore, despite earlier evidence of preserved LVEF, male rats on the fructose high salt diet display a deficit in myocardial function as evidenced by reduced GLS. In contrast, female rats are protected from the cardiovascular derangements caused by the fructose and high salt diet as they remain normotensive with aortic PWV and GLS comparable to those measured in their control counterparts. The present study is consistent with female rats being protected from the impact of combined fructose and high salt on blood pressure, aortic stiffness, and early left ventricular dysfunction.

## AUTHOR CONTRIBUTIONS

*Conception and design*: Noreen F. Rossi. *Performed, analyzed and interpretation of data*: Dragana Komnenov and Noreen F. Rossi. *Drafted manuscript*: Dragana Komnenov. *Revised and edited manuscript*: Noreen F. Rossi. *Approved final draft*: Dragana Komnenov and Noreen F. Rossi.

## ETHICS STATEMENT

Ethics approval was granted by the Institutal Animal Care and Use Committee (IACUC 20‐04‐2085). All animal work adhered to the protocols as delineated in the approval. The datasets analyzed during the current study are available from the corresponding author on reasonable request.

## FUNDING INFORMATION

This work was supported by grants HL163844 from the National Institutes of Health RX000851 from the U.S. Department of Veterans Affairs and to NFR.

## CONFLICT OF INTEREST STATEMENT

The authors have no conflicts of interest to declare.
